# A tale of three next generation sequencing platforms: comparison of Ion Torrent, Pacific Biosciences and Illumina MiSeq sequencers

**DOI:** 10.1186/1471-2164-13-341

**Published:** 2012-07-24

**Authors:** Michael A Quail, Miriam Smith, Paul Coupland, Thomas D Otto, Simon R Harris, Thomas R Connor, Anna Bertoni, Harold P Swerdlow, Yong Gu

**Affiliations:** 1 Wellcome Trust Sanger Institute, Hinxton, UK

**Keywords:** Next-generation sequencing, Ion torrent, Illumina, Pacific biosciences, MiSeq, PGM, SMRT, Bias, Genome coverage, GC-rich, AT-rich

## Abstract

**Background:**

Next generation sequencing (NGS) technology has revolutionized genomic and genetic research. The pace of change in this area is rapid with three major new sequencing platforms having been released in 2011: Ion Torrent’s PGM, Pacific Biosciences’ RS and the Illumina MiSeq. Here we compare the results obtained with those platforms to the performance of the Illumina HiSeq, the current market leader. In order to compare these platforms, and get sufficient coverage depth to allow meaningful analysis, we have sequenced a set of 4 microbial genomes with mean GC content ranging from 19.3 to 67.7%. Together, these represent a comprehensive range of genome content. Here we report our analysis of that sequence data in terms of coverage distribution, bias, GC distribution, variant detection and accuracy.

**Results:**

Sequence generated by Ion Torrent, MiSeq and Pacific Biosciences technologies displays near perfect coverage behaviour on GC-rich, neutral and moderately AT-rich genomes, but a profound bias was observed upon sequencing the extremely AT-rich genome of *Plasmodium falciparum* on the PGM, resulting in no coverage for approximately 30% of the genome. We analysed the ability to call variants from each platform and found that we could call slightly more variants from Ion Torrent data compared to MiSeq data, but at the expense of a higher false positive rate. Variant calling from Pacific Biosciences data was possible but higher coverage depth was required. Context specific errors were observed in both PGM and MiSeq data, but not in that from the Pacific Biosciences platform.

**Conclusions:**

All three fast turnaround sequencers evaluated here were able to generate usable sequence. However there are key differences between the quality of that data and the applications it will support.

## Background

Sequencing technology is evolving rapidly and during the course of 2011 several new sequencing platforms were released. Of note were the Ion Torrent Personal Genome Machine (PGM) and the Pacific Biosciences (PacBio) RS that are based on revolutionary new technologies.

The Ion Torrent PGM “harnesses the power of semiconductor technology” detecting the protons released as nucleotides are incorporated during synthesis [1]. DNA fragments with specific adapter sequences are linked to and then clonally amplified by emulsion PCR on the surface of 3-micron diameter beads, known as Ion Sphere Particles. The templated beads are loaded into proton-sensing wells that are fabricated on a silicon wafer and sequencing is primed from a specific location in the adapter sequence. As sequencing proceeds, each of the four bases is introduced sequentially. If bases of that type are incorporated, protons are released and a signal is detected proportional to the number of bases incorporated.

PacBio have developed a process enabling single molecule real time (SMRT) sequencing [2]. Here, DNA polymerase molecules, bound to a DNA template, are attached to the bottom of 50 nm-wide wells termed zero-mode waveguides (ZMWs). Each polymerase is allowed to carry out second strand DNA synthesis in the presence of γ-phosphate fluorescently labeled nucleotides. The width of the ZMW is such that light cannot propagate through the waveguide, but energy can penetrate a short distance and excite the fluorophores attached to those nucleotides that are in the vicinity of the polymerase at the bottom of the well. As each base is incorporated, a distinctive pulse of fluorescence is detected in real time.

In recent years, the sequencing industry has been dominated by Illumina, who have adopted a sequencing-by-synthesis approach [3], utilizing fluorescently labeled reversible-terminator nucleotides, on clonally amplified DNA templates immobilized to an acrylamide coating on the surface of a glass flowcell. The Illumina Genome Analyzer and more recently the HiSeq 2000 have set the standard for high throughput massively parallel sequencing, but in 2011 Illumina released a lower throughput fast-turnaround instrument, the MiSeq, aimed at smaller laboratories and the clinical diagnostic market.

Here we evaluate the output of these new sequencing platforms and compare them with the data obtained from the Illumina HiSeq and GAIIx platforms. Table 1 gives a summary of the technical specifications of each of these instruments.

**Table 1:** Technical specifications of Next Generation Sequencing platforms utilised in this study

**Platform**	**Illumina MiSeq**	**Ion Torrent PGM**	**PacBio RS**	**Illumina GAIIx**	**Illumina HiSeq 2000**
Instrument Cost*	$128 K	$80 K**	$695 K	$256 K	$654 K
Sequence yield per run	1.5-2Gb	20-50 Mb on 314 chip, 100-200 Mb on 316 chip, 1Gb on 318 chip	100 Mb	30Gb	600Gb
Sequencing cost per Gb*	$502	$1000 (318 chip)	$2000	$148	$41
Run Time	27 hours***	2 hours	2 hours	10 days	11 days
Reported Accuracy	Mostly > Q30	Mostly Q20	<Q10	Mostly > Q30	Mostly > Q30
Observed Raw Error Rate	0.80 %	1.71 %	12.86 %	0.76 %	0.26 %
Read length	up to 150 bases	~200 bases	Average 1500 bases**** (C1 chemistry)	up to 150 bases	up to 150 bases
Paired reads	Yes	Yes	No	Yes	Yes
Insert size	up to 700 bases	up to 250 bases	up to 10 kb	up to 700 bases	up to 700 bases
Typical DNA requirements	50-1000 ng	100-1000 ng	~1 μg	50-1000 ng	50-1000 ng

* All cost calculations are based on list price quotations obtained from the manufacturer and assume expected sequence yield stated.

** System price including PGM, server, OneTouch and OneTouch ES.

*** Includes two hours of cluster generation.

**** Mean mapped read length includes adapter and reverse strand sequences. Subread lengths, i.e. the individual stretches of sequence originating from the sequenced fragment, are significantly shorter.

## Results

### Sequence generation

Platform specific libraries were constructed for a set of microbial genomes *Bordetella pertussis* (67.7% GC, with some regions in excess of 90% GC content), *Salmonella* Pullorum (52% GC), *Staphylococcus aureus* (33% GC) and *Plasmodium falciparum* (19.3% GC, with some regions close to 0% GC content). We routinely use these to test new sequencing technologies, as together their sequences represent the range of genomic landscapes that one might encounter.

PCR-free [4] Illumina libraries were uniquely barcoded, pooled and run on a MiSeq flowcell with paired 150 base reads plus a 6-base index read and also on a single lane of an Illumina HiSeq with paired 75 base reads plus an 8-base index read (Additional file 1: Table S1). Illumina libraries prepared with amplification using Kapa HiFi polymerase [5] were run on a single lane of an Illumina GA IIx with paired 76 base reads plus an 8-base index read and on a MiSeq flowcell with paired 150 base reads plus a 6-base index read. PCR-free libraries represent an improvement over the standard Illumina library preparation method as they result in more even sequence coverage [4] and are included here alongside libraries prepared with PCR in order to enable comparison to PacBio which has an amplification free workflow.

Ion Torrent libraries were each run on a single 316 chip for a 65 cycles generating mean read lengths of 112–124 bases (Additional file 1: Table S2). Standard PacBio libraries, with an average of 2 kb inserts, were run individually over multiple SMRT cells, each using C1 chemistry, and providing ≥20x sequence coverage data for each genome (Additional file 1: Table S3).

The datasets generated were mapped to the corresponding reference genome as described in Methods. For a fair comparison, all sequence datasets were randomly down-sampled (normalized) to contain reads representing a 15x average genome coverage.

### Workflow

All the platforms have library preparation protocols that involve fragmenting genomic DNA and attaching specific adapter sequences. Typically this takes somewhere between 4 and 8 hours for one sample. In addition, the Ion Torrent template preparation has a two hour emulsion PCR and a template bead enrichment step.

In the battle to become the platform with the fastest turnaround time, all the manufacturers are seeking to streamline library preparation protocols. Life Technologies have developed the Ion Xpress Fragment Library Kit that has an enzymatic “Fragmentase” formulation for shearing starting DNA, thereby avoiding the labour of physical shearing and potentially enabling complete library automation. We tested this kit on our four genomes alongside the standard library kit with physical shearing and found both to give equal genomic representation (see Additional file 2: Figure S1 for results obtained with *P. falciparum*). Illumina purchased Epicentre in order to package the Nextera technology with the MiSeq. Nextera uses a transposon to shear genomic DNA and simultaneously introduce adapter sequences [6]. The Nextera method can produce sequencing ready DNA in around 90 minutes and gave us remarkably even genome representation (Additional file 2: Figures S2 and Additional file 2: Figure S3) with *B. pertussis* and *S. aureus*, but produced a very biased sequence dataset from the extremely AT-rich *P. falciparum* genome.

### Genome coverage and GC bias

**Figure 1:**
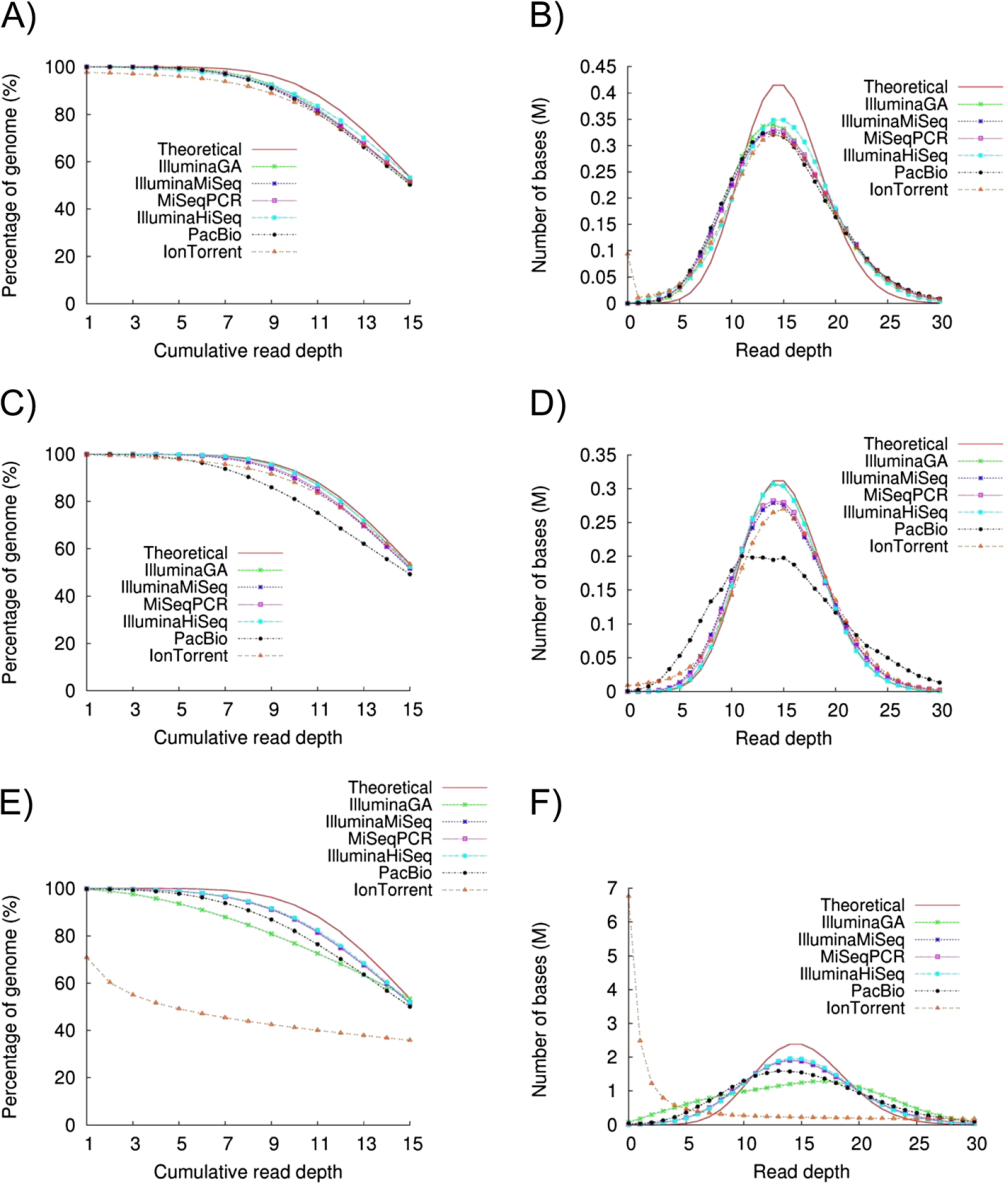
**Genome coverage plots for 15x depth randomly downsampled sequence coverage from the sequencing platforms tested. A**) The percentage of the *B. pertussis* genome covered at different read depths; **B**) The number of bases covered at different depths for *B. pertussis;***C**) The percentage of the *S. aureus* genome covered at different read depths; **D**) The number of bases covered at different depths for *S. aureus;***E**) The percentage of the *P. falciparum* genome covered at different read depths; and **F**) The number of bases covered at different depths for *P. falciparum.*

To analyse the uniformity of coverage across the genome we tabulated the depth of coverage seen at each position of the genome. We utilized the coverage plots described by Lam et al., [7] that depict; the percentage of the genome that is covered at a given read depth, and genome coverage at different read depths respectively, for each dataset (Figure 1) alongside the ideal theoretical coverage that would be predicted based on Poisson behaviour.

In the context of the GC-rich genome of *B. pertussis*, most platforms gave similar uniformity of sequence coverage, with the Ion Torrent data giving slightly more uneven coverage. In the *S. aureus* genome the PGM performed better. The PGM gave very biased coverage when sequencing the extremely AT-rich *P. falciparum* genome (Figure 1). This affect was also evident when we plotted coverage depth against GC content (Additional file 2: Figure S4). Whilst the PacBio platform gave a sequence dataset with quite even coverage on GC and extremely AT-rich contexts, it did demonstrate slight but noticeable unevenness of coverage and bias towards GC-rich sequences with the *S. aureus* genome. With the GC-neutral *S.* Pullorum genome all platforms gave equal coverage with unbiased GC representation (data not shown).

**Figure 2:**
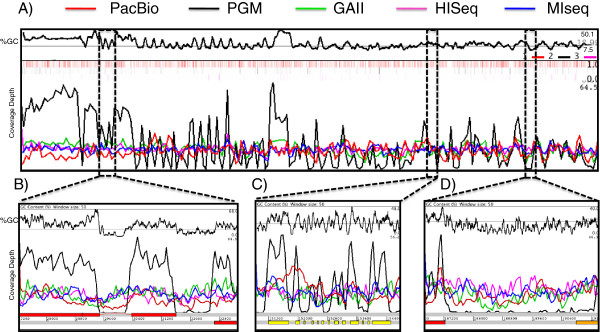
**Artemis genome browser**[8]**screenshots illustrating the variation in sequence coverage of a selected region of *P. falciparum *chromosome 11, with 15x depth of randomly normalized sequence from the platforms tested.** In each window, the top graph shows the percentage GC content at each position, with the numbers on the right denoting the minimum, average and maximum values. The middle graph in each window is a coverage plot for the dataset from each instrument; the colour code is shown above graph a). Each of the middle graphs shows the depth of reads mapped at each position, and below that in B-D are the coordinates of the selected region in the genome with gene models on the (+) strand above and (−) strand below. **A**) View of the first 200 kb of chromosome 11. Graphs are smoothed with window size of 1000. A heatmap of the errors, normalized by the amount of mapping reads is included just below the GC content graph (PacBio top line, PGM middle and MiSeq bottom). **B**) Coverage over region of extreme GC content, ranging from 70% to 0%. **C**) Coverage over the gene PF3D7_1103500. **D**) Example of intergenic region between genes PF3D7_1104200 and PF3D7_1104300. The window size of B, C and D is 50 bp.

The most dramatic observation from our results was the severe bias seen when sequencing the extremely AT-rich genome of *P. falciparum* on the PGM. The result of this was deeper than expected coverage of the GC-rich *var* and subtelomeric regions and poor coverage within introns and AT-rich exonic segments (Figure 2), with approximately 30% of the genome having no sequence coverage whatsoever. This bias was observed with libraries prepared using both enzymatic and physical shearing (Additional file 2: Figure S1).

**Figure 3:**
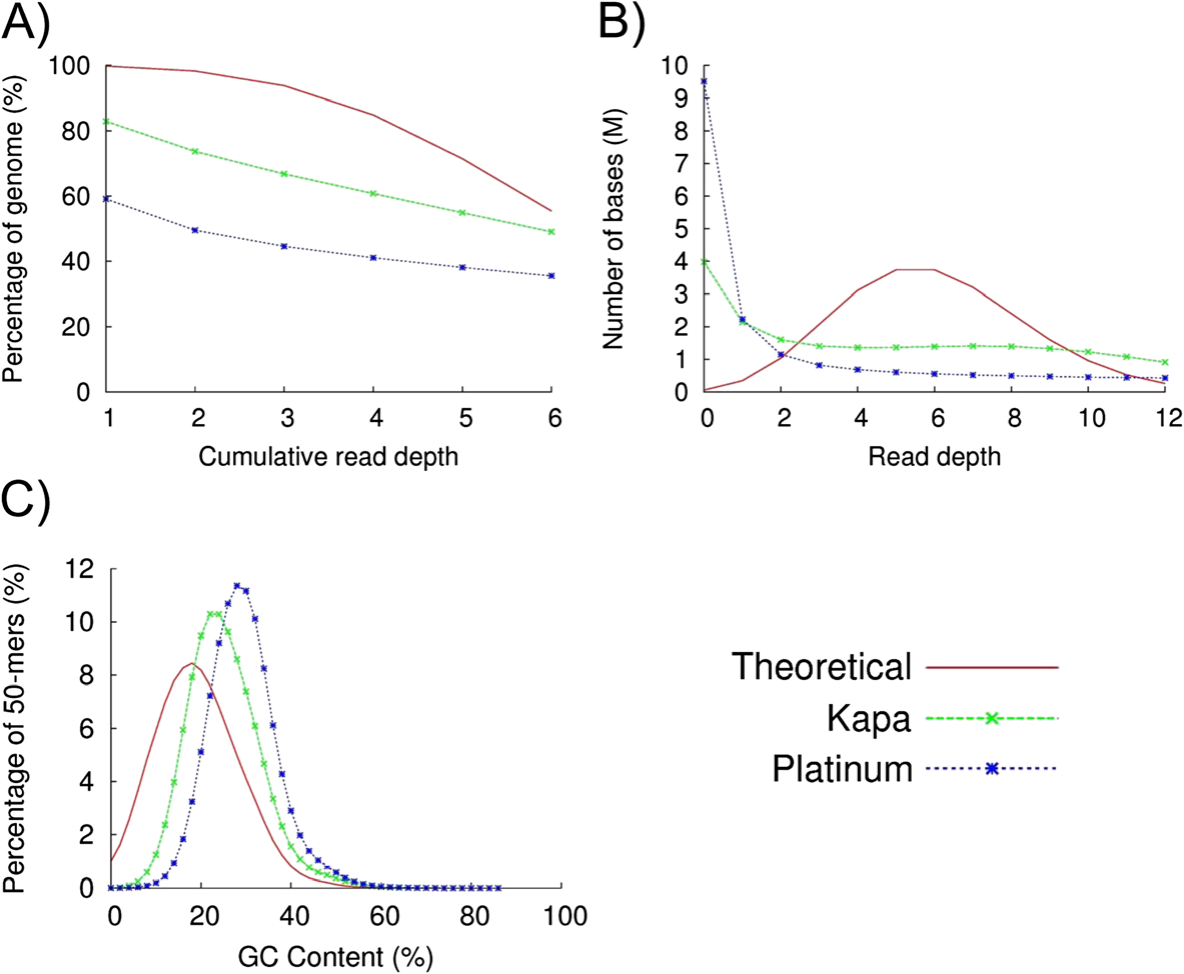
**The effect of substituting Platinum HiFi PCR supermix with Kapa HiFi in the PGM library prep amplification step. A**) The percentage of the *P. falciparum* genome covered at different read depths. The blue line shows the data obtained with the recommended Platinum enzyme and the green line with Kapa HiFi. The red line depicts ideal coverage behavior. **B**) The number of bases covered at different depths. **C**) Sequence representation vs. GC-content plots.

In a recent study to investigate the optimal enzyme for next generation library preparation [5], we found that the enzyme used for fragment amplification during next generation library preparation can have a significant influence on bias. We found the enzyme Kapa HiFi amplifies fragments with the least bias, giving even coverage, close to that obtained without amplification. Since the PGM has two amplification steps, one during library preparation and the other emulsion PCR (emPCR) for template amplification, we reasoned that this might be the cause of the observed bias. Substituting the supplied Platinum Taq enzyme with Kapa HiFi for the nick translation and amplification step during library preparation profoundly reduced the observed bias (Figure 3). We were unable to further improve this by use of Kapa HiFi for the emPCR (results not shown).

Of the four genomes sequenced, the *P. falciparum* genome is the largest and most complex and contains a significant quantity of repetitive sequences. We used *P. falciparum* to analyse the effect of read length versus mappability. As the PacBio pipeline doesn’t generate a mapping quality value and to ensure a fair comparison, we remapped the reads of all technologies using the k-mer based mapper, SMALT [9], and then analysed coverage across the *P. falciparum* genome (Additional file 3: Table S4). This data confirms the poor performance of Ion Torrent on the *P. falciparum* genome, as only 65% of the genome is covered with high quality (>Q20) reads compared to ~98-99% for the other platforms. Whilst the mean mapped readlength of the PacBio reads with this genome was 1336 bases, average subread length (the length of sequence covering the genome) is significantly less (645 bases). The short average subread length is due to preferential loading of short fragment constructs in the library and the effect of lag time (non-imaged bases) after sequencing initiation, the latter resulting in sequences near the beginning of library constructs not being reported.

As the median length of the PacBio subreads for this data set are just 600 bases, we compared their coverage with an equivalent amount of *in silico* filtered reads of >620 bases. This led to a very small decrease in the percentage of bases covered. Using paired reads on the Illumina MiSeq, however, gave a strong positive effect, with 1.1% more coverage being observed from paired-end reads compared to single-end reads.

### Error rates

**Figure 4:**
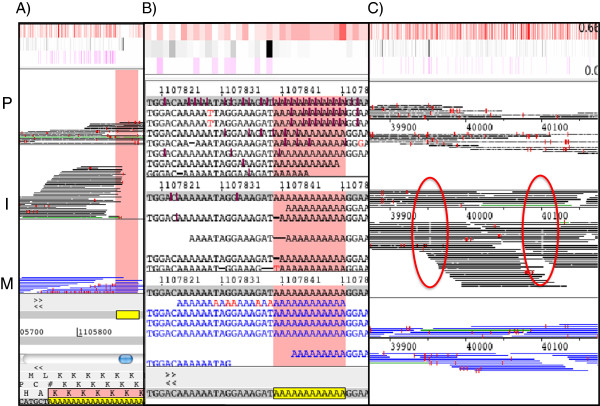
**Illustration of platform-specific errors.** The panels show Artemis BAM views with reads (horizontal bars) mapping to defined regions of chromosome 11 of *P. falciparum* from PacBio (P; top), Ion Torrent (I; middle) and MiSeq (M; bottom). Red vertical dashes are 1 base differences to the reference and white points are indels. **A**) Illustration of errors in Illumina data after a long homopolymer tract. Ion torrent data has a drop of coverage and multiple indels are visible in PacBio data. **B**) Example of errors associated with short homopolymer tracts. Multiple insertions are visible in the PacBio Data, deletions are observed in the PGM data and the MiSeq sequences read generally correct through the homopolymer tract. **C**) Example of strand specific deletions (red circles) observed in Ion Torrent data.

We observed error rates of below 0.4% for the Illumina platforms, 1.78% for Ion Torrent and 13% for PacBio sequencing (Table 1). The number of error-free reads, without a single mismatch or indel, was 76.45%, 15.92% and 0% for, MiSeq, Ion Torrent and PacBio, respectively. The error heatmap in Figure 2A shows that the PacBio errors are distributed evenly over the chromosome. We manually inspected the regions where Ion Torrent and Illumina generated more errors. Illumina produced errors after long (> 20-base) homopolymer tracts [10] (Figure 4A).

Also evident in the MiSeq data, were strand errors due to the GGC motif [11]. Following the finding that the motif GGC generates strand-specific errors, we analyzed this phenomenon in the MiSeq data for *P. falciparum* (Additional file 4: Table S5). We observed that the error is mostly generated by GC-rich motifs, principally GGCGGG. We found no evidence for an error if the triplet after the GGC is AT-rich. Other MiSeq datasets also showed this artifact (data not shown). In addition to this being a strand-specific issue, it appears that this is a read-specific phenomenon. Whilst there is a quality drop in the first read following these GC-rich motifs, there is a striking loss of quality in read 2, where the reads have nearly half the mean quality value compared to the read 1 reads for GC-rich triplets that follow the GGC motif. We could observe this low quality in read 2 in all our analysed Illumina lanes. For AT-rich motifs the ratio is nearly 1 (1.03).

Ion Torrent didn’t generate reads at all for long (> 14-base) homopolymer tracts, and could not predict the correct number of bases in homopolymers >8 bases long. Very few errors were observed following short homopolymer stretches in the MiSeq data (Figure 4B). Additionally, we observed strand-specific errors in the PGM data but were unable to associate these with any obvious motif (Figure 4C).

### SNP calling

**Figure 5:**
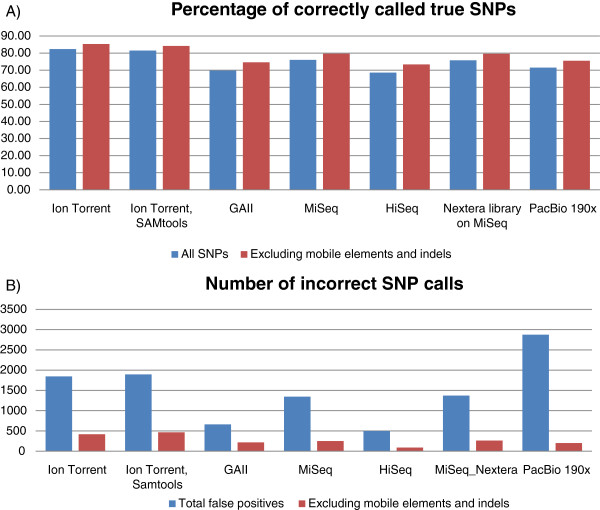
**Accuracy of SNP detection from the *S. aureus *datasets generated from each platform, compared against the reference genome of its close relative*S. aureus* USA300_FPR3757.** Both the Torrent server variant calling pipeline and SAMtools were used for Ion Torrent data; SAMtools was used for Illumina data and SMRT portal pipeline for PacBio data. **A**) The percentage of SNPs detected using each platform overall (blue bar), and outside of repeats, indels and mobile genetic elements (red bar). **B**) The number of incorrect SNP calls for each platform overall (blue bar), and outside of repeats, indels and mobile genetic elements (red bar).

In order to determine whether or not the higher error rates observed with the PGM and PacBio affected their ability to call SNPs, we aligned the reads from the *S. aureus* genome, for which all platforms gave good sequence representation, against the reference genome of the closely related strain USA300_FPR3757 [12]*,* and compared the SNPs called against those obtained by aligning the reference sequences of the two genomes (Figure 5 and Additional file 5: Table S6). In order to create a fair comparison we initially used the same randomly normalized 15x datasets used in our analysis of genome coverage, which according to the literature [3] is sufficient to accurately call heterozygous variants but found that that was insufficient for the PacBio datasets where a 190x coverage was used.

Overall the rate of SNP calling was slightly higher for the Ion Torrent data than for Illumina data (chi square p value 3.15E-08), with approximately 82% of SNPs being correctly called for the PGM and 68-76% of the SNPs being detected from the Illumina data (Figure 5A). Conversely, the rate of false SNP calls was higher with Ion Torrent data than for Illumina data (Figure 5B). SNP calling from PacBio data proved more problematic, as existing tools are optimized for short-read data and not for high error-rate long-read data. We were reliant on SNPs called by the SMRT portal pipeline for this analysis. Our results showed that SNP detection from PacBio data was not as accurate as that from the other platforms, with overall only 71% of SNPs being detected and 2876 SNPs being falsely called (Additional file 5: Table S6).

Amongst the datasets obtained from the Illumina sequencers, the percentage of correct SNP calls was higher for the MiSeq (76%) than the GAIIx (70%) data than for that obtained from the HiSeq (69%), despite the same libraries being run on both MiSeq and HiSeq. The use of Nextera library preparation gave similar results with 76% of SNPs being correctly called. It should be noted that we found the inbuilt automatic variant calling inadequate on both MiSeq and PGM, with MiSeq reporter calling just 6.6% of variants and Torrent suite 1.5.1 calling only 1.4% of variants.

## Discussion

A key feature of these new platforms is their speed. Decreasing run time has clear advantages particularly within the clinical sequencing arena, but poses challenges in itself. Whilst manufacturers may state library prep times on the order of a couple of hours, these times don’t include upfront QC and library QC and quantification. Also, typical library prep times quoted usually apply to processing of only one sample; i.e., pipetting time is largely ignored. Purchasers of sequencing instruments will want to keep them running at full utilization, in order to maximize their investment and will also want to pool multiple samples on single runs for economic reasons. To obtain maximum throughput, users must consider the whole process, potentially investing in ancillary equipment and robotics to create an automated pipeline for the preparation of large numbers of samples. To process large numbers of samples quickly, a facility’s instrument base must be large enough to avoid sample backlogs. With this in mind, manufacturers are seeking to develop more streamlined sample-prep protocols that will facilitate timely sample loading. Here we have tested two such developments: enzymatic fragmentation and the Nextera technique. We conclude that these methods can be very useful, but users must carefully evaluate the methods they use for their particular applications and for use with genomes of extreme base composition to avoid bias.

Whilst the data generated using the Ion Torrent PGM platform has a higher raw error rate (~1.8%) than Illumina data (<0.4%), provided there is sufficient coverage, the representation and ability to call SNPs is quite closely matched between these technologies with more true positives being called from PGM data but far less false positives from the Illumina data. Detection of SNPs using PacBio data was not as accurate; the use of single-molecule sequencing to detect low level variants and quasispecies within populations remains unproven. We have found PacBio’s long reads useful for scaffolding *de novo* assemblies, though our experience suggests that this is currently not fully optimized and extensive method development is still required.

Interestingly, the mappability didn’t increase significantly with longer reads, although a beneficial effect was obtained from having mate-pair information. Current PacBio protocols favor the preferential loading of smaller constructs, resulting in average subread lengths that are significantly shorter than the often quoted average read lengths. Further development is therefore required to avoid having excess short fragments and adapter-dimer constructs in the library and reducing their loading efficiency into the ZMWs.

Whilst one would normally use higher coverage than used here for confident SNP detection (i.e., 30-40x depth), we were limited to 15x depth due to the yield of some of the platforms. Nonetheless, at least for the haploid genome, *S*. *aureus*, 15x coverage should be a reasonable quantity for SNP detection and even in the human genome, 15x coverage has been shown to be sufficient to accurately call heterozygous SNPs [3].

Variant calling is a highly subjective process; the particular software chosen as well as the specific parameters employed to make the predictions will change the results substantially. As such, the rate of both true SNP and false positive calling provided here are purely indicative and results obtained with each sequencing platform will vary. For any particular application using a specific sequencing method, optimisation of the SNP- and indel-calling algorithm would always be recommended.

We sequence many isolates of the malaria parasite *P. falciparum* as it represents a significant health issue in developing countries; this organism leads to several million deaths per annum. There are several active large sequencing programs (e.g. MalariaGEN [13]) that are currently aiming to sequence thousands of clinical malaria samples. As the malaria genome has a GC content of only 19.4% [14], we use it as one of our test genomes, representing a significant challenge to most sequencing technologies. Based on the present study, use of Illumina sequencing technology with libraries prepared without amplification [4] leads to the least biased coverage across this genome. Ion Torrent semiconductor sequencing is not recommended for sequencing of extremely AT-rich genomes, due to the severe coverage bias observed. This is likely to be an artifact introduced during amplification. Therefore, avoidance of library amplification and/or emPCR, or use of more faithful enzymes during emPCR, may eliminate the bias.

Illumina sequencing has matured to the point where a great many applications [15-24] have been developed on the platform. Since the PGM is also a massively parallel short-read technology, many of those applications should translate well and be equally practicable. There are a few obvious exceptions; techniques involving manipulations on the flow cell such as FRT-seq [21] and OS-Seq [22] will be impossible using semiconductor sequencing. Also, the Ion Torrent platform currently employs fragment lengths of 100 or 200 bases; without a mate-pair type library protocol, these insert sizes are too short perhaps to enable accurate *de novo* assemblies such as that demonstrated using ALLPATHS-LG for mammalian genomes using Illumina data [25]. Conversely, Illumina sequencing on the HiSeq or MiSeq instruments requires heterogeneous base composition across the population of imaged clusters [26]. In order to sequence monotemplates (where most sequenceable fragments have exactly the same sequence), it is often necessary to significantly dilute or mix the sample with a complex genomic library to enable registration of clusters. Semiconductor sequencing does not suffer this problem.

The DNA-input requirements of PacBio can be prohibitory. Illumina and PGM library preparation can be performed with far less DNA; the standard PGM IonEXpress library prep requires just 100 ng DNA and Illumina sequencing has been performed from sub-nanogram quantities [27]. The yield, sample-input requirements and amplification-free library prep of PacBio potentially make it unsuitable for counting applications and for applications involving significant prior enrichment such as exome sequencing [15] and ChIP-seq [18]. The PacBio platform, by virtue of its long read lengths, should however have application in de novo sequencing and may also benefit analysis of linkage of alternative splicing and in of variants across long amplicons. Furthermore, the potential for direct detection of epigenetic modifications has been demonstrated [28].

Finally, it should be noted that thus study represents a point in time, utilising kits and reagents available up until the end of 2011. Ion Torrent and Pacific Biosciences are relatively new sequencing technologies that have not had time to mature in the same way that the Illumina technology has. We anticipate that whilst some of the issues identified may be intrinsic, others will be resolved as these platforms evolve.

## Conclusion

The limited yield and high cost per base currently prohibit large scale sequencing projects on the Pacific Biosciences instrument. The PGM and MiSeq are quite closely matched in terms of utility and ease of workflow. The decision on whether to purchase one or the other will hinge on available resources, existing infrastructure and personal experience, available finances and the type of applications being considered.

## Methods

### Genomic DNA

*P. falciparum* 3D7 genomic DNA was a gift from Prof Chris Newbold, University of Oxford, UK. *Bordetella pertussis* ST24 genomic DNA was a gift from Craig Cummings, Stanford University School of Medicine, CA. *Staphylococcus aureus* TW20 genomic DNA was a gift from Jodi Lindsay, St George’s Hospital Medical School, University of London. *S.* Pullorum S449/87 genomic DNA was prepared at the Wellcome Trust Sanger Institute, UK.

### Illumina library construction

DNA (0.5 μg in 120 μl of 10 mM Tris–HCl pH8.5) was sheared in an AFA microtube using a Covaris S2 device (Covaris Inc.) with the following settings: Duty cycle 20, Intensity 5, cycles/burst 200, 45 seconds.

Sheared DNA was purified by binding to an equal volume of Ampure beads (Beckman Coulter Inc.) and eluted in 32 μl of 10 mM Tris–HCl, pH8.5. End-repair, A-tailing and paired-end adapter ligation were performed (as per the protocols supplied by Illumina, Inc. using reagents from New England Biolabs- NEB) with purification using a 1.5:1 ratio of standard Ampure to sample between each enzymatic reaction. PCR-free libraries were constructed according to Kozarewa et al. [4]. After ligation, excess adapters and adapter dimers were removed using two Ampure clean-ups, first with a 1.5:1 ratio of standard Ampure to sample, followed by a 0.7:1 ratio of Ampure beads. PCR free libraries were then used as is. Libraries prepared with amplification were diluted to 2 ng/μl and 1 μl was used as template for PCR amplification with Kapa HiFi [5] 2 x mastermix (KK2601, Kapa Biosystems). PCR reactions were performed in 0.2 μl thin-wall microtubes on an MJ tetrad thermal cycler with the following conditions: 94°C −2 minutes; 14 cycles of 94°C −20 seconds, 65°C −30 seconds, 72°C −30 seconds; 72°C - 3 minutes with 200nM final concentration of standard PE1.0 and modified multiplexing PE2.0 primers [5].

After PCR, excess primers and any primer dimer were removed using two Ampure clean-ups, with a 1.5:1 ratio of Ampure then with a 0.8:1 ratio of Ampure beads. All libraries were quantified by real-time PCR using the SYBR Fast Illumina Library Quantification Kit (Kapa Biosystems) and pooled so as to give equal genome coverage from each library.

### Illumina sequencing

Each multiplexed library pool was sequenced on: i) an Illumina GAIIx instrument for 76 cycles from each end plus an 8 base-index sequence read, using version 2 chemistry, ii) an Illumina MiSeq for 151 cycles from each end plus an 8 base-index sequence read, iii) an Illumina HiSeq 2000 instrument for 75 cycles from each end plus an 8 base-index sequence read, using version 3 chemistry. Summary sequencing statistics are given in Additional file 1: Table S1. All runs had error rates, and associated sequence quality, that surpassed the minimum Illumina specifications.

### Ion torrent library preparation sequencing

For the *B. pertussis*, *S. aureus* and *P. falciparum* genomes, library preparation was carried out using the Ion Xpress™ Fragment Library Kit, with 100 ng of DNA. Adapter ligation, size selection, nick repair and amplification (8 cycles for *B. pertussis* and *S. aureus*, 6 cycles for *P. falciparum*) were performed as described in the Ion Torrent protocol associated with the kit (Ion Xpress™ Fragment Library Kit - Part Number 4469142 Rev. B). For the *S.* Pullorum genome, library preparation was undertaken using the Ion Fragment Library Kit with 5 μg of DNA. The DNA was fragmented by adaptive focused acoustics using a Covaris S2 (Covaris Inc.) with AFA tubes as described in the protocol (Part Number 4467320 Rev. A). End repair, adapter ligation, nick repair and amplification (8 cycles) were also performed as described in the Ion Torrent protocol. Size selection was performed using the LabChip XT (Caliper LifeSciences) and the LabChip XT DNA750 Assay Kit (Caliper LifeSciences), with collection between 175 bp and 220 bp.

The Agilent 2100 Bioanalyzer (Agilent Technologies) and the associated High Sensitivity DNA kit (Agilent Technologies) were used to determine quality and concentration of the libraries. The amount of library required for template preparation was calculated using the Template Dilution Factor calculation described in the protocol.

Emulsion PCR and enrichment steps were carried out using the Ion Xpress™ Template Kit and associated protocol (Part Number 4469004 Rev. B). Ion Sphere Particle quality assessment was carried out as outlined in an earlier version of this protocol (Part Number 4467389 Rev. B) for all samples because no benefit was seen with using the Ion Sphere Quality Control Kit as recommended in the later version of the protocol. The oligos used for this analysis were purchased from IDT (Integrated DNA Technologies Inc.). Assessment of the Ion Sphere Particle quality was undertaken between the emulsion PCR and enrichment steps only.

### Ion torrent sequencing

Sequencing was undertaken using 316 chips in all cases and barcoding was not used for these samples. The Ion Sequencing Kit v2.0 was used for all sequencing reactions, following the recommended protocol (Part Number 4469714 Rev. B) and Torrent Suite 1.5 was used for all analyses. Summary sequencing statistics are given in Additional file 1: Table S2.

### PacBio library construction

DNA (2.0-10 μg in 200 μl 10 mM Tris–HCl pH8.5) was sheared in an AFA clear mini-tube using a Covaris S2 device (Covaris Inc.) with the following settings: Duty cycle 20, Intensity 0.1, cycles/burst 1000, 600 seconds. Sheared DNA was purified by binding to 0.6X volume of pre-washed AMPure XP beads (Beckman Coulter Inc.), as per PacBio protocol 000-710-821-DRAFT (five times in purified water, one time in EB, reconstituted in original supernatant) and eluted in EB to >60 ng/μl. The sheared DNA was quantified on an Agilent 2100 Bioanalyzer using the 7500 kit. 1 μg of sheared DNA was end-repaired using the PacBio DNA Template Prep Kit 1.0 (Part Number 001-322-716) and incubated for 15 min at 25°C prior to another 0.6X AMPure XP clean up, eluting in 30 μl EB. Blunt adapters were ligated before exonuclease incubation was carried out in order to remove all un-ligated adapters and DNA. Finally, two 0.6X AMPure bead clean ups are performed - removing enzymes and adapter dimers – the final “SMRT bells” being eluted in 10 μl EB. Final quantification was carried out on an Agilent 2100 Bioanalyzer with 1 μl of library.

Using the SMRT bell concentration (ng/μl) and insert size previously determined, the PacBio-provided calculator was used to calculate the amounts of primer and polymerase used for the binding reaction. Using the PacBio DNA/Polymerase Binding Kit 1.0 (Part Number 001-359-802), primers are annealed and the proprietary polymerase is bound forming the “Binding Complex”. The Binding Complex can be stored as a long-term storage mix at −20°C or diluted for immediate sequencing. The quantity of SMRT bell determines whether a long-term storage mix can be used. In this instance, there was ample genomic DNA from the four test genomes to allow long-term storage.

### PacBio sequencing

Long-term storage mixes were diluted to the required concentration and volume with the provided dilution buffer and loaded into 96-well plates. These are loaded onto the instrument, along with DNA Sequencing Kit 1.0 (Part Number 001-379-044) and a SMRT Cell 8Pac. In all sequencing runs, 2x45 min movies were captured for each SMRT Cell loaded with a single binding complex. Primary filtering analysis was performed on the Blade Center server provided with the RS instrument, before this data was transferred off the Blade Center for secondary analysis in SMRT Portal using the SMRT analysis pipeline version 1.2.0.1.81002. Summary sequencing statistics are given in Additional file 1: Table S3.

### Reference genomes

Each reference genome was created using capillary sequence data with manual finishing and are available to download from http://www.sanger.ac.uk/resources/downloads/. The methods used to sequence the genomes of *P. falciparum*[14] and *S. aureus* TW20 [29] have been published.

### Data processing

After sequencing, reads were mapped to each genome reference sequence using the manufacturers’ alignment tools, tmap for PGM and blasr for PacBio (http://www.pacificbiosciences.com/products/software/algorithms). BWA [30] was used for mapping reads from the Illumina GAIIx, MiSeq and HiSeq. SAMtools [31] was then used to generate pileup and coverage information from the mapping output.

### Genome coverage

We counted the number of bases in the genome that were not covered by any reads (Coverage = 0) and those with less than 5x read coverage (Coverage <5x). SAMtools was used to generate coverage plots and bash/awk scripts were used for coverage counting.

### Evenness of coverage metrics

We extracted genome coverage information from the pileup data derived by SAMtools from mapped reads after randomly down sampling to a uniform depth of 15x (this down sampling was achieved by taking “from the top” the number of reads required to give 15x coverage of each genome). As reads are randomly allocated evaluation of uniformity of coverage was based on cumulative distributions over the overall average depth.

### GC-content analysis

To evaluate the coverage uniformity in different genome regions, a GC profile was calculated for each data set. All mapped reads were shredded into 50-mers and the GC-percentage in each 50-mer was calculated. The proportions of 50-mers containing a given GC-percentage were plotted against their GC percentage. A theoretical curve for each genome was also produced in the same way from its reference sequence for comparison. The difference from the theoretical curve gives an indication of GC bias.

### Alignment base error analysis

The aligned error rate for data generated on the different sequencing platforms was taken from the report generated by the program SMALT [9], after aligning the *S. aureus* dataset against its reference sequence. The error rate is calculated as the per-base error within a mapped region divided by the total mapped bases in that region. An average error rate was calculated from all mapped reads for each data set.

To quantify errors associated with specific motifs, we took the fastq file and searched all the reads for the presence of that motif. The three bases (triplets) after the motif were tabulated, and the mean quality of the following base was calculated. We did this analysis for GGC, GCC and a neutral motif - ATG.

### SNP detection

SNP detection was performed using a random selection of reads to give an average depth of coverage of 15x for all platforms, except PacBio where this coverage depth was insufficient and the full dataset representing 190x coverage was used.

SNPs from the PacBio reads were called using PacBio’s SMRT Portal software version 1.2.3. Each SMRT cell was selected for analysis against the *S. aureus* USA300_FPR3757 reference genome (accession number CP000255), imported into the PacBio secondary analysis protocol; the parameters can be altered for filtering, mapping, and consensus calling. SFilter.1.xml was used for filtering with a minimum allowed read length of 50 bases and a minimum read quality of 0.75 (on a PacBio-developed scale specific to RS-generated reads). BLASR.1.xml was used for mapping with the maximum number of hits per read being set to 1, a maximum divergence of 30% and minimum anchor size of 8. Finally, EviCons.1.xml was used for consensus and SNP calling. Reads from the Illumina and Ion Torrent platforms were mapped against the *S. aureus* USA300_FPR3757 reference using SMALT [9].

SNPs were called using the default parameters for SAMtools mpileup followed by bcftools and the SAMtools vcfutils.pl varFilter script, as described on the SAMtools webpage (http://samtools.sourceforge.net/mpileup.shtml). SNPs were also called for the Ion Torrent data using the Torrent Suite variant calling parameters for SAMtools mpileup and bcftools followed by the Torrent Suite vcf_filter.pl script.

A set of reference SNPs was created by aligning the complete *S. aureus* USA300_FPR3757 genome sequence with a high-quality draft sequence for *S. aureus* TW20 using Mugsy [32]. A single contiguous whole-genome alignment was generated by extracting aligned blocks from the Mugsy output and then manually curating. In order to control for the effects of software-specific mis-mapping, we identified and removed from our alignment regions sequences corresponding to mobile genetic elements (MGEs) in the *S. aureus* USA300_FPR3757 genome, along with regions with no homologue in *S. aureus*. MGEs were manually identified from the *S. aureus* USA300_FPR3757 genome annotation SNPs called from the resulting alignment provided a high-quality reference set for comparison with the SNPs identified by each sequencing platform. True SNPs are those that agree with the SNPs found in this reference set.

All datasets have been deposited in the ENA read archive under accession number ERP001163.

## Abbreviations

NGS, Next-generation sequencing; PGM, Personal genome machine; SMRT, Single molecule real time; PCR, Polymerase chain reaction; emPCR, Emulsion PCR; PE, Paired-end; qPCR, Quantitative PCR; QC, Quality Control; SNP, Single nucleotide polymorphism; Q10, 1 error in 10; Q20, 1 error in 100; Q30, 1 error in 1000.

## Competing interests

The authors declare no competing financial interests.

## Authors’ contributions

MQ, MS, PC and AB performed the experiments and performed primary data analysis. MQ, MS, PC and HPS designed the experiments. MQ wrote the manuscript. TDO, YGU, SH and TC carried out bioinformatics analysis. All authors read and approved the final manuscript.

## Supplementary Material

Additional file 1: Table S1 Statistics for Illumina Sequencing Runs. **Table S2.** Statistics for Ion Torrent Sequencing Runs. **Table S3.** Statistics for PacBio Sequencing Runs. Click here for file

Additional file 2: Figure S1 Comparison of the outcome of sequencing using libraries prepared using enzymatic shearing (green line) and physical shearing (blue line) on the Ion Torrent PGM. A) The percentage of the P*. falciparum* genome covered at different read depths; B) The number of bases covered at different depths; C) Sequence representation versus GC content. **Figure S2.** Genome coverage uniformity plots for 15x depth randomly normalized sequence coverage from sequencing libraries prepared using standard and Nextera Library preparation methods. A) The percentage of the *B. pertussis* genome covered at different read depths; B) The number of bases covered at different depths for *B. pertussis;* C) The percentage of the *S. aureus* genome covered at different read depths; D) The number of bases covered at different depths for *S. aureus;* E) The percentage of the *P. falciparum* genome covered at different read depths; and F) The number of bases covered at different depths for *P. falciparum*. **Figure S3.** Sequence representation versus GC content for 15x depth randomly normalized sequence coverage from sequencing libraries prepared using standard and Nextera Library preparation methods. Genome coverage uniformity plots for 15x depth randomly normalized sequence coverage from sequencing libraries prepared using the Illumina Nextera Library preparation kit (blue line) compared to those prepared using a standard Illumina library preparation with Kapa HiFi for library amplification (green line), on: A) *B. pertussis*; B) *S. aureus* and C) *P. falciparum* genomes. **Figure S4.** Sequence representation versus GC content for 15x depth randomly normalized sequence coverage from the sequencing platforms tested, on: A) *B. pertussis*; B) and C) *P. falciparum* genomes. Click here for file

Additional file 3: Table S4 Comparison of sequence coverage for data generated with PacBio, PGM and MiSeq across the P. falciparum genome. Reads from randomly normalized 15x datasets were remapped with SMALT to have a uniform mapping score. To analyse the utility of long reads, read length and mate-pair read analysis was also performed on 15x datasets comprising PacBio reads longer than 620 bases, and MiSeq paired- and single-end datasets with 150-base, 100-base and 50-base read lengths. Click here for file

Additional file 4: Table S5 Ratios of the occurrence of quality loss after specific sequence triplets following the GGC motif. For each strand, the occurrence and subsequent mapping quality is tabulated for the GGC motif and for comparison another GC-rich motif GCC and the neutral motif ATG. Ratios are then given for the sequence quality observed on the forward and reverse strands following the GGC triplet and ratios of mapping quality on the same strand following GCC and ATG triplets when compared to the GGC triplet. Click here for file

Additional file 5: Table S6 SNP detection statistics for *S. aureus* datasets versus *S. aureus* USA300_FPR3757. Click here for file
