# Supplementary material for: A tale of three next generation sequencing platforms: comparison of Ion Torrent, Pacific Biosciences and Illumina MiSeq sequencers
Source: BMC Genomics. 2012 Jul 24;13:341. doi: 10.1186/1471-2164-13-341 (PMC3431227; doi:10.1186/1471-2164-13-341)
Supplement: Additional file 1: Table S1 — Statistics for Illumina Sequencing Runs. Table S2. Statistics for Ion Torrent Sequencing Runs. Table S3. Statistics for PacBio Sequencing Runs. [file 1471-2164-13-341-S1.doc]

|  |  |  |  |
| --- | --- | --- | --- |
| **Table S1: Statistics for Illumina Sequencing Runs** | | | |
|  |  |  |  |
|  |  |  |  |
|  |  |  |  |
| **GAIIx** |  |  |  |
|  |  |  |  |
| **Genome** | ***B. pertussis*** | ***S. aureus*** | ***P. falciparum*** |
|  |  |  |  |
| Number of Reads | 6102346 | 2036618 | 52503290 |
| Read Length | 76 | 76 | 76 |
| Number of duplicates | 33873 | 17148 | 780691 |
| Mapped yield | 391.11 Mb | 151.14 Mb | 3884.6 Mb |
| Coverage | 97.3 | 48.62 | 166.72 |
|  |  |  |  |
| Cluster density | 226K clusters/tile |  |  |
|  |  |  |  |
|  |  |  |  |
| **HiSeq** |  |  |  |
|  |  |  |  |
| **Genome** | ***B. pertussis*** | ***S. aureus*** | ***P. falciparum*** |
|  |  |  |  |
| Number of Reads | 54996906 | 46283212 | 49738806 |
| Read Length | 75 | 76 | 76 |
| Number of duplicates | 1424919 | 610067 | 492747 |
| Mapped yield | 3980.62 Mb | 3426.15 Mb | 3640.94 Mb |
| Coverage | 990.2 | 1102.36 | 156.26 |
|  |  |  |  |
| Cluster density | 382K clusters/tile |  |  |
|  |  |  |  |
|  |  |  |  |
| **MiSeq noPCR** |  |  |  |
|  |  |  |  |
| **Genome** | ***B. pertussis*** | ***S. aureus*** | ***P. falciparum*** |
|  |  |  |  |
| Number of Reads | 3183730 | 2940258 | 3334062 |
| Read Length | 151 | 151 | 151 |
| Number of duplicates | 3146 | 1482 | 2868 |
| Mapped yield | 327.6 Mb | 421.4 Mb | 464.12 Mb |
| Coverage | 81.4 | 135.59 | 19.9 |
|  |  |  |  |
| Cluster density | 876K clusters/mm2 |  |  |
|  |  |  |  |
|  |  |  |  |
|  |  |  |  |
|  |  |  |  |
| **MiSeq PCR** |  |  |  |
|  |  |  |  |
| **Genome** | ***B. pertussis*** | ***S. aureus*** | ***P. falciparum*** |
|  |  |  |  |
| Number of Reads | 3957274 | 3625240 | 4122588 |
| Read Length | 151 | 151 | 151 |
| Number of duplicates | 7962 | 2671 | 2077 |
| Mapped yield | 454 Mb | 520.6 Mb | 574.4 Mb |
| Coverage | 112.9 | 167.5 | 24.6 |
|  |  |  |  |
| Cluster density | 1067K clusters/mm2 |  |  |
|  |  |  |  |
|  |  |  |  |
| **MiSeq Nextera** |  |  |  |
|  |  |  |  |
| **Genome** | ***B. pertussis*** | ***S. aureus*** | ***P. falciparum*** |
|  |  |  |  |
| Number of Reads | 1988770 | 2036618 | 2578342 |
| Read Length | 151 | 151 | 151 |
| Number of duplicates | 9219 | 17148 | 4582 |
| Mapped yield | 225.02 Mb | 284.73 Mb | 353.52 Mb |
| Coverage | 55.98 | 91.61 | 15.17 |
|  |  |  |  |
| Cluster density | 786K clusters/mm2 |  | |

| **Table S2: Statistics for Ion Torrent PGM Sequencing Runs** | | | | |
| --- | --- | --- | --- | --- |
|  |  |  |  |  |
|  |  |  |  |  |
|  |  |  |  |  |
|  |  |  |  |  |
| **Genome** | ***B. pertussis*** | ***S. aureus*** | ***P. falciparum*** | ***P. falciparum* Kapa HiFi** |
|  |  |  |  |  |
| Chip | 316 | 316 | 316 | 316 |
| Number of Runs | 1 | 1 | 4 | 1 |
| Total Number of Bases | 283.51Mb | 324.28 Mb | 868.08 Mb | 220.3 Mb |
| Number of Q17 bases | 204.29 Mb | 238.38 Mb | 618.57 Mb | 129.45 Mb |
| Number of Q20 bases | 174.84 Mb | 205.61 Mb | 533.24 Mb | 97.61 Mb |
| Number of Reads | 2464690 | 2716711 | 7169172 | 1959564 |
| Mean length | 115 | 119 | 121 | 112 |
| Longest read | 201 | 203 | 203 | 203 |
| Mean Q17 coverage depth | 48.60 | 75.90 | 22.90 | 5.00 |
|  |  |  |  |  |

| **Table S3: Statistics for PacBio Sequencing Runs** | | |  |
| --- | --- | --- | --- |
|  |  |  |  |
|  |  |  |  |
|  | ***P. falciparum*** | ***S. aureus*** | ***B. pertussis*** |
|  |  |  |  |
| % Adapter Dimer (0-10bp) | 0.33 | 0.02 | 0.27 |
| % Short Insert (11-100bp) | 0.43 | 0.10 | 0.60 |
| # of SMRT Cells | 25 | 18 | 11 |
| # of Movies | 50 | 36 | 21 |
| Mean Depth of Coverage | 0.85 | 15.04 | 9.05 |
| % Missing Bases | 58.84 | 0.01 | 0.44 |
| # of Post-Filter Reads | 21310.36 | 67336.67 | 57067.45 |
| # of Mapped Reads | 18330.76 | 44275.17 | 40337.36 |
| # of Mapped Bases | 25335180.88 | 66193419.67 | 47585399.18 |
| 95th Percentile Mapped Readlength | 3160.88 | 3497.44 | 3042.73 |
| Maximum Mapped Readlength | 5374.88 | 5942.11 | 5605.45 |
| Mean Mapped Readlength | 1336.64 | 1493.50 | 1185.64 |
| # of Mapped Subreads | 33002.68 | 83307.22 | 63381.55 |
| Mean Mapped Subread Readlength | 645.72 | 614.33 | 653.27 |
| Median Mapped Subread Readlength | 600 | 567 | 535 |
| Mean Mapped Subread Accuracy | 84.94 | 84.11 | 83.76 |
| Coverage | 21.32 | 270.67 | 99.55 |
