# Supplementary figures and images for: A tale of three next generation sequencing platforms: comparison of Ion Torrent, Pacific Biosciences and Illumina MiSeq sequencers
Source: BMC Genomics. 2012 Jul 24;13:341. doi: 10.1186/1471-2164-13-341 (PMC3431227; doi:10.1186/1471-2164-13-341)

## Slide 1
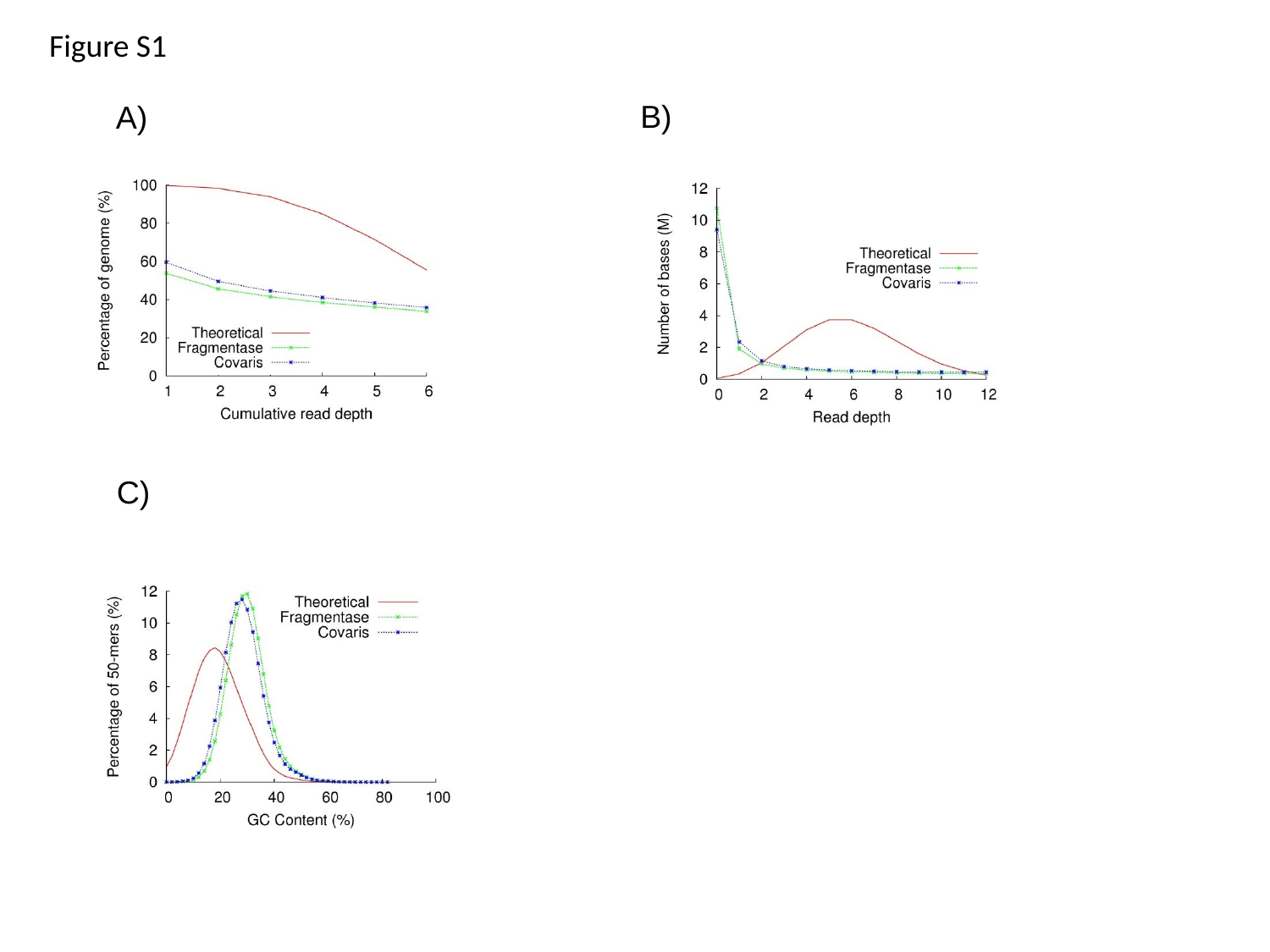

Figure S1
B)
A)
C)

## Slide 2
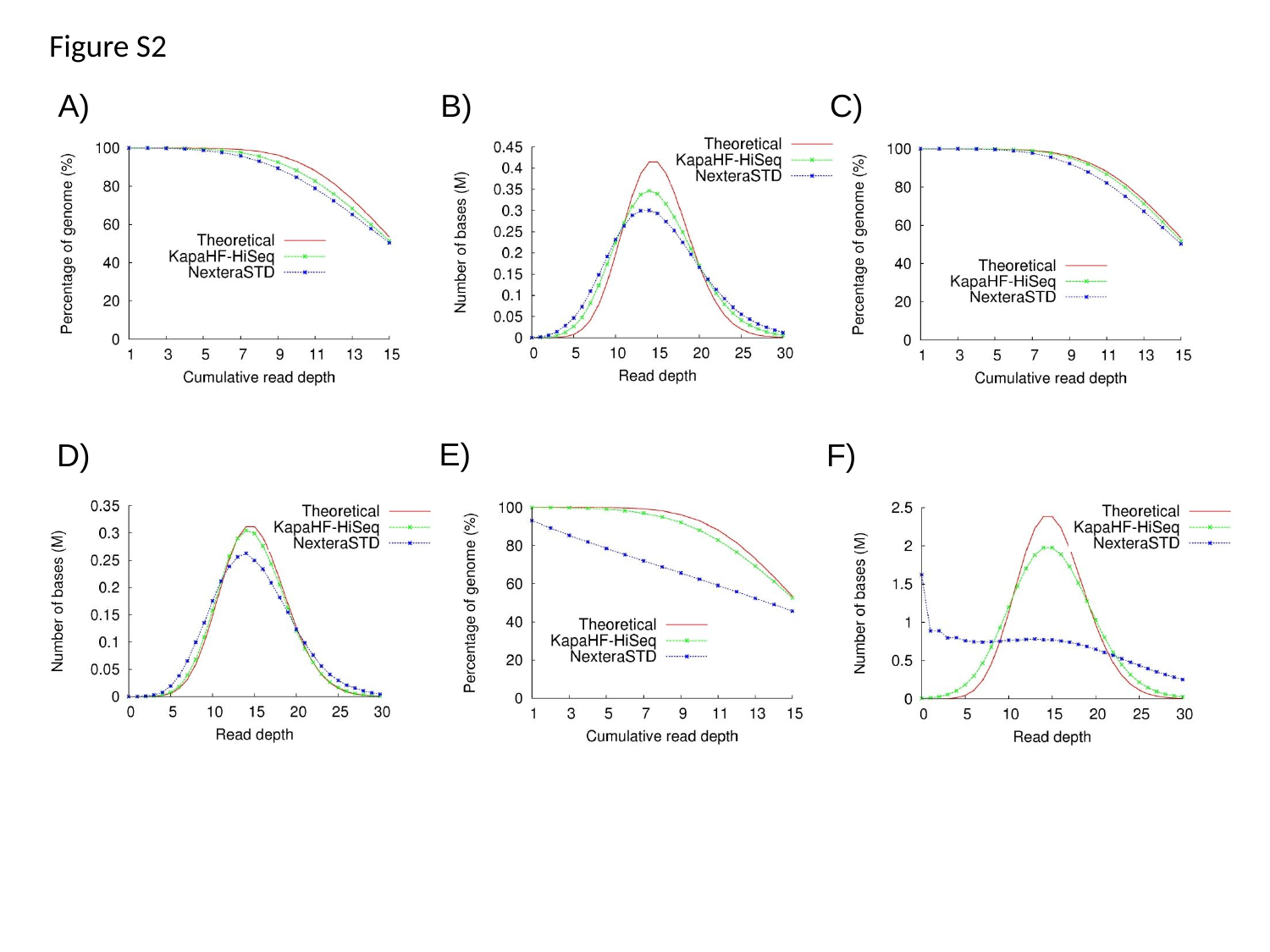

Figure S2
B)
A)
C)
E)
D)
F)

## Slide 3
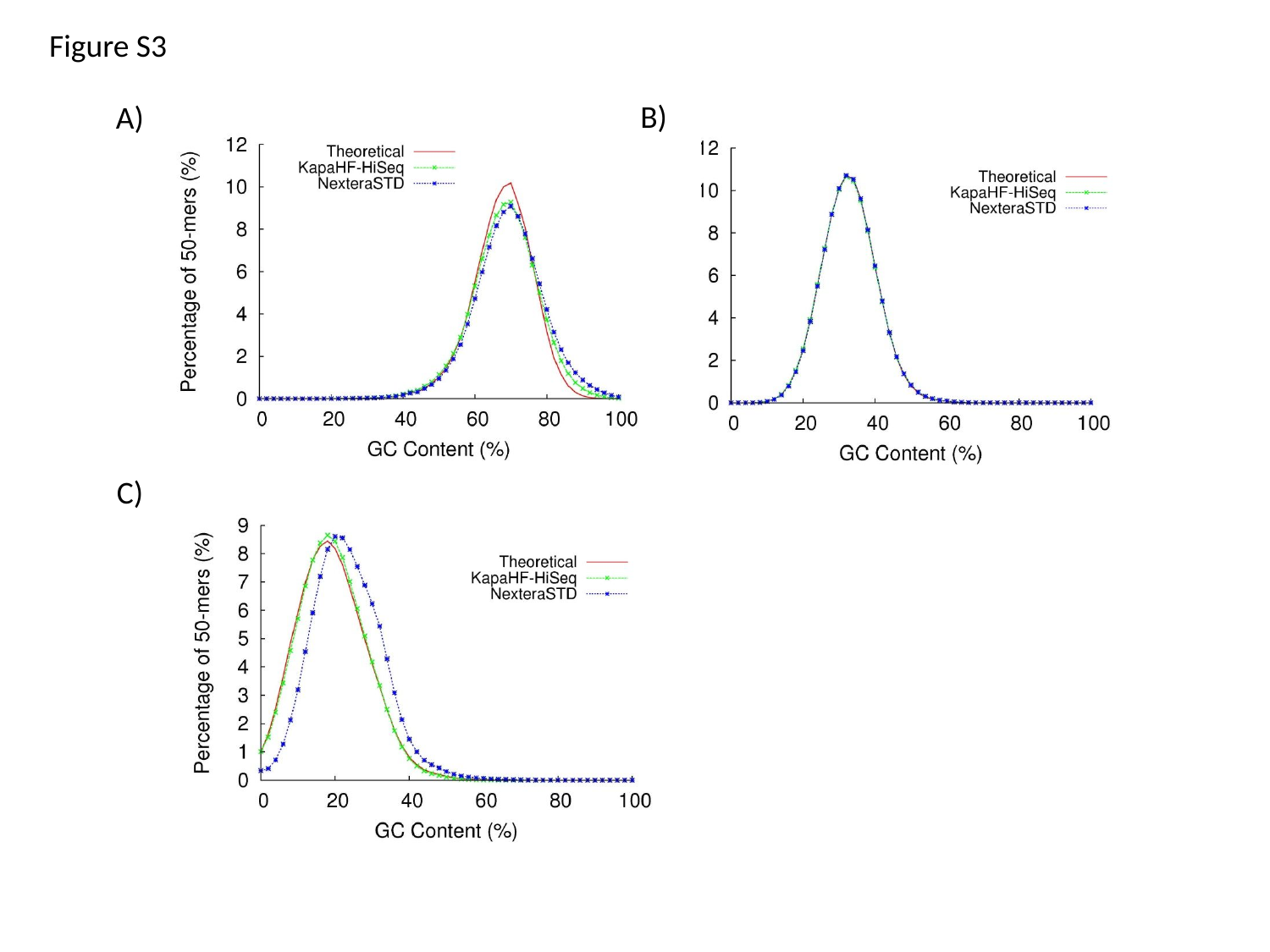

Figure S3
B)
A)
C)

## Slide 4
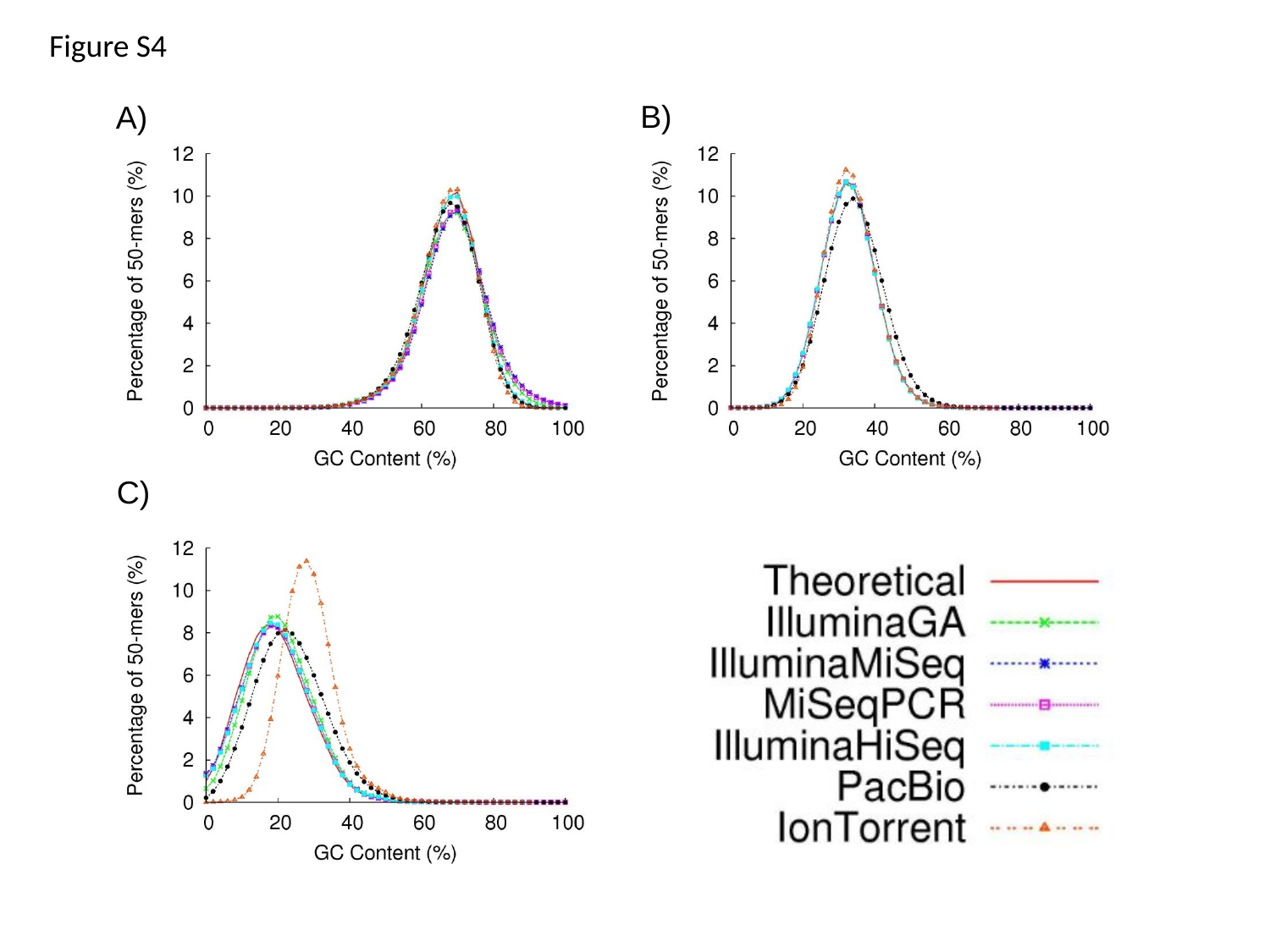

Figure S4
B)
A)
C)

Supplement: Additional file 2: Figure S1 — Comparison of the outcome of sequencing using libraries prepared using enzymatic shearing (green line) and physical shearing (blue line) on the Ion Torrent PGM. A) The percentage of the P. falciparum genome covered at different read depths; B) The number of bases covered at different depths; C) Sequence representation versus GC content. Figure S2. Genome coverage uniformity plots for 15x depth randomly normalized sequence coverage from sequencing libraries prepared using standard and Nextera Library preparation methods. A) The percentage of the B. pertussis genome covered at different read depths; B) The number of bases covered at different depths for B. pertussis; C) The percentage of the S. aureus genome covered at different read depths; D) The number of bases covered at different depths for S. aureus; E) The percentage of the P. falciparum genome covered at different read depths; and F) The number of bases covered at different depths for P. falciparum. Figure S3. Sequence representation versus GC content for 15x depth randomly normalized sequence coverage from sequencing libraries prepared using standard and Nextera Library preparation methods. Genome coverage uniformity plots for 15x depth randomly normalized sequence coverage from sequencing libraries prepared using the Illumina Nextera Library preparation kit (blue line) compared to those prepared using a standard Illumina library preparation with Kapa HiFi for library amplification (green line), on: A) B. pertussis; B) S. aureus and C) P. falciparum genomes. Figure S4. Sequence representation versus GC content for 15x depth randomly normalized sequence coverage from the sequencing platforms tested, on: A) B. pertussis; B) and C) P. falciparum genomes. [file 1471-2164-13-341-S2.ppt]
